# A year in the life of *eLife*

**DOI:** 10.7554/eLife.01516

**Published:** 2013-10-15

**Authors:** Randy Schekman, Fiona M Watt, Detlef Weigel

**Keywords:** scientific publishing, publishing, peer review, elife, open access

## Abstract

Improving the peer review process, overcoming the limitations of print journals and providing open access to the very best work in the life and biomedical sciences are three highlights of our first year.

Complaining about journals is a popular topic whenever scientists meet. Scientific journals are too slow, and they reject too many papers. Referees ask for too many revisions, and editors let them get away with it. Authors are obsessed with impact factors, and the peer review process—the process that is meant to keep standards high—seems close to breaking point. A letter published in *Science* in 2008 said it all: ‘The stress associated with publishing experimental results—a process that can take as long as obtaining the results in the first place—can drain much of the joy from practicing science’ (Raff et al., 2008). *eLife* was set up to address many of these issues and now, one year after we published our first papers, is a good time to reflect on the progress we have made.

A new approach to peer review is one of the foundations on which *eLife* is built. The review process at any journal begins with editors who initially assess the work. Compared to academic editors, professional editors have fewer distractions, but they also often lack the in-depth knowledge that is needed to properly assess many of the manuscripts that they handle. This has four major drawbacks. First, simply deciding whether a manuscript should be sent to external referees for in-depth peer review can take weeks. Second, after all the referee reports have been received, they are often passed on to the author with relatively little guidance from the editor on how the manuscript needs to be revised to maximize its chances of acceptance. Third, revised manuscripts are automatically sent back to the referees in many cases, regardless of whether or not the referees have indicated that they are willing to take part in a second round of review. Finally, it is not unusual for more than one round of revision to be required: this means that the process can drag on for months and months, with no guarantee that the manuscript will be accepted (Snyder, 2013). In the meantime, publication of similar results by other authors, often in journals with less strict standards, may lead to rejection of the original manuscript, despite all the efforts made by the original authors to address the criticisms levelled by the referees.

At *eLife*, we have overcome many of these difficulties by changing several aspects of the traditional peer review process (Schekman et al., 2013). Each manuscript submitted to the journal is assigned to a Senior Editor who decides—often after consultation with one or more members of the Board of Reviewing Editors—whether or not it merits in-depth peer review. On average, we tell authors within just three days whether we are interested in sending their manuscript to referees for detailed review. With 20 leading researchers working as Senior Editors, and another 170 serving as Reviewing Editors, *eLife* has access to expertise in almost every area of the life sciences, from systems neurobiology and structural biology to medical genetics and ecological genomics.

If the manuscript is selected for in-depth peer review, one of the Reviewing Editors typically acts as a referee and takes the lead in identifying one or two other referees. After all the referee reports have been received, the Reviewing Editor, with support from a Senior Editor, oversees an online discussion of the strengths and weaknesses of the manuscript under review. This happens in a blog-like format on a secure website where the referees are identified to each other and communicate as peers over the course of a day or more; in some cases there have been more than 30 discussion comments! This open process ensures that a referee will be held accountable in a discussion with known colleagues. If the consensus is that the work should be published in *eLife*, the main points of the referee reports are distilled into a single decision letter that clearly spells out what additional analysis, if any, is required before the manuscript can be accepted: in general we only ask for essential revisions that will not unduly delay publication. Even with this additional back-and-forth between the referees, on average it takes only 28 days to return a decision to the authors. However, the real benefits of this extra work are seen at the next stage of the process: most manuscripts that are accepted have undergone just one round of revision, with the process of revision and re-evaluation taking an average of just six weeks. All these figures are on our website.

Most manuscripts that are accepted have undergone just one round of revision.

Compared to other journals, the *eLife* process requires a modest amount of additional effort from the referees. However, this is balanced to some extent by *eLife* preferring to make decisions based on just two or three referee reports (including the report from the Reviewing Editor). In the interests of transparency, we encourage authors to endorse publication of the most substantive parts of the decision letter, and their responses, along with the main article.

Taking full advantage of the opportunities offered by digital media constitutes the second pillar of the *eLife* approach. Because there are no print issues, we do not arbitrarily limit the number of words, figures and references in an article. We can also seamlessly integrate movies into the text, and link underlying data sets to their graphical summary representation. This is complemented by eLife Lens, a new way of dynamically displaying scientific articles online, instead of merely replicating the print version (Figure 1). Based on ideas developed by Ivan Grubisic, a graduate student at UC Berkeley, *eLife Lens* allows readers to move between the text, figures and references in a way that is more intuitive and flexible than existing approaches to online journals. We have also dispensed with the artificial separation between Printed figures and supplementary figures, and are pleased that we are no longer alone with these efforts.

**Figure 1.**
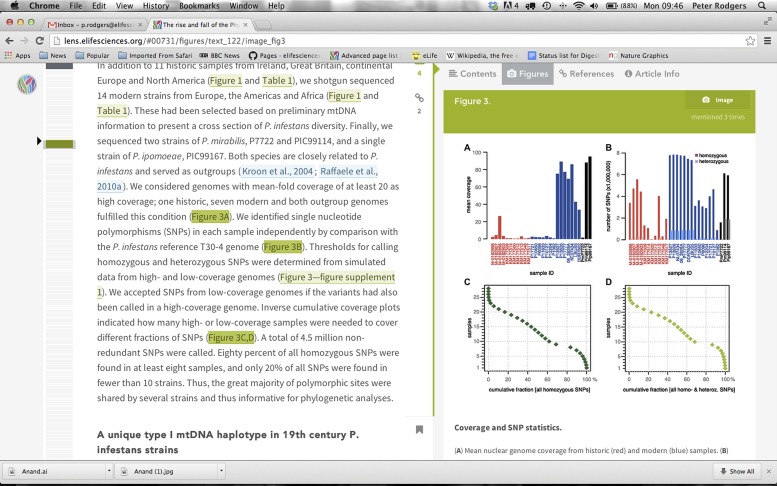
*eLife* takes full advantage of the opportunities offered by digital media. A new article viewer, eLife Lens, allows the reader to move intuitively between the text, figures and references of an article.

Because there are no print issues, we do not arbitrarily limit the number of words, figures and references in an article.

In addition to giving authors enough words, figures and references to tell their story without having to relegate important aspects of it to supplementary information, the online-only approach means that we can publish all manuscripts that meet our standards. The acceptance rates of many other journals, on the other hand, still seem to be influenced by page budgets that date back to the print era: such page budgets are an artificially restricted commodity in the age of electronic publication. This problem is particularly acute at some of the most highly competitive print journals. Submitting manuscripts to these journals can be likened to applying for a job at Google: because so many excellent candidates apply, Google does not worry about how many of these excellent candidates are rejected. Its main concern is that everyone who makes it through the interview process should fit the Google mould. Scientific journals should not work in this way, with only a limited number of vacancies. At *eLife* we aim to publish work of a certain standard, and we accept all manuscripts that reach or exceed this standard. Taking the Google approach, on the other hand, inevitably leads to too many excellent papers being rejected by the most selective journals. Related to this we helped to draft the San Francisco Declaration on Research Assessment because we feel strongly that research needs to be assessed on its own merits, not on where it was published (Vale, 2012; Vosshall, 2012; Schekman and Patterson, 2013).

Our third main motivation for *eLife* has been to reinvigorate the push for open access in scientific publication, so that research that has been overwhelmingly funded from taxes or not-for-profit sources is freely available. While there has been a recent proliferation of open access journals, many publishers still put profit before service to the biomedical community and the wider public, so there is still some way to go on this front.

As a service to authors and readers, each article is accompanied by an *eLife* digest, which summarizes in 300–400 words the essence of an article in language that should be readily understandable to anyone with an interest in science. Moreover, *eLife* does not impose an embargo once a paper has been accepted for publication, and authors are free to communicate their findings to colleagues, as well as the media and the public, by posting preprints on their own website or in a repository or preprint server. Several of our authors have made excellent use of such opportunities, and a number of *eLife* papers have been covered in the popular media, including the BBC, the *New York Times* and *Scientific American*, and in other scientific periodicals, such as *Cell*, *Nature* and *Science*.

Beyond our research articles, we select a subset of papers for in-depth commentaries called Insights, and we have published Feature Articles on topics as diverse as funding for biomedical research in the US and a Facebook game that allows members of the public to help with genomics research. And in an on-going series of Living Science essays, *eLife* Senior Editor Eve Marder discusses the challenges of squaring a scientific career with having a satisfying personal life.

*eLife* was founded by three of the most important non-governmental funders of biomedical research, the Howard Hughes Medical Institute, the Max Planck Society and the Wellcome Trust, all of whom apply extremely strict criteria in evaluating their scientists. To ensure the success of *eLife*, the three founders have committed almost £15m to the launch and development of the journal, but editorial control rests entirely with the editors. Further details of *eLife* operations can be found in the first annual report.

So, is *eLife* working? Yes, it is. In the first twelve months, we have already published more than 180 research articles on a wide range of subjects, from the origins of multicellularity in animals (which was literally our first paper) and plants performing arithmetic so that they do not run out of starch by morning, to the evolution of cancer after targeted combination therapy and the discovery of the Hepatitis B receptor. Notably, from the very beginning, the average quality of submissions has been very high, with only a small minority not meriting any discussion at all among the editors.

While the three of us felt some trepidation when we agreed to devote a significant amount of our time to *eLife*, we were united in our conviction that the publication landscape was in dire need of change. We were not alone in this view, and were able to recruit a fantastic team of editors. Our combined efforts are being richly rewarded by the great manuscripts that researchers are submitting to *eLife* and by the enthusiastic feedback we are receiving from authors, referees and readers. Our publishing activities are only the beginning of a great journey: please join us in making *eLife* a journal that is truly designed for science at its very best.
